# Trait Expression and Environmental Responses of Pea (*Pisum sativum* L.) Genetic Resources Targeting Cultivation in the Arctic

**DOI:** 10.3389/fpls.2021.688067

**Published:** 2021-07-29

**Authors:** Ulrika Carlson-Nilsson, Karolina Aloisi, Ingunn M. Vågen, Ari Rajala, Jørgen B. Mølmann, Søren K. Rasmussen, Mari Niemi, Ewelina Wojciechowska, Pertti Pärssinen, Gert Poulsen, Matti W. Leino

**Affiliations:** ^1^NordGen, Nordic Genetic Resource Center, Alnarp, Sweden; ^2^Division of Food Production and Society, Norwegian Institute of Bioeconomy Research NIBIO, Ås, Norway; ^3^Natural Resources Institute Finland (Luke), Jokioinen, Finland; ^4^Department of Plant and Environmental Sciences, University of Copenhagen, Frederiksberg, Denmark; ^5^Boreal Plant Breeding Ltd., Jokioinen, Finland; ^6^Danish Seed Savers, Tjele, Denmark; ^7^The Archaeological Research Laboratory, Stockholm University, Stockholm, Sweden

**Keywords:** phenology, phenotyping, ideotype, yield components, thermal modeling, garden pea, field pea, landraces

## Abstract

In the Arctic part of the Nordic region, cultivated crops need to specifically adapt to adverse and extreme climate conditions, such as low temperatures, long days, and a short growing season. Under the projected climate change scenarios, higher temperatures and an earlier spring thaw will gradually allow the cultivation of plants that could not be previously cultivated there. For millennia, Pea (*Pisum sativum* L.) has been a major cultivated protein plant in Nordic countries but is currently limited to the southern parts of the region. However, response and adaptation to the Arctic day length/light spectrum and temperatures are essential for the productivity of the pea germplasm and need to be better understood. This study investigated these factors and identified suitable pea genetic resources for future cultivation and breeding in the Arctic region. Fifty gene bank accessions of peas with a Nordic landrace or cultivar origin were evaluated in 2-year field trials at four Nordic locations in Denmark, Finland, Sweden, and Norway (55° to 69° N). The contrasting environmental conditions of the trial sites revealed differences in expression of phenological, morphological, crop productivity, and quality traits in the accessions. The data showed that light conditions related to a very long photoperiod partly compensated for the lack of accumulated temperature in the far north. A critical factor for cultivation in the Arctic is the use of cultivars with rapid flowering and maturation times combined with early sowing. At the most extreme site (69°N), no accession reached full maturation. Nonetheless several accessions, predominantly landraces of a northern origin, reached a green harvest state. All the cultivars reached full maturation at the sub-Arctic latitude in northern Sweden (63°N) when plants were established early in the season. Seed yield correlated positively with seed number and aboveground biomass, but negatively with flowering time. A high yield potential and protein concentration of dry seed were found in many garden types of pea, confirming their breeding potential for yield. Overall, the results indicated that pea genetic resources are available for breeding or immediate cultivation, thus aiding in the northward expansion of pea cultivation. Predicted climate changes would support this expansion.

## Introduction

Agriculture in the Arctic part of the Nordic region requires crops that have adapted to extreme climate conditions, such as low temperatures, very long days, and a short growing season. Today, the variety of these crops is more or less limited to forage crops and barley, but climate change scenarios project it will be possible to cultivate more crops in these areas in the near future. Of particular interest are crops that can provide plant protein for both livestock and humans. An increased cultivation of grain legumes is already considered suitable in Europe, in general, and in northernmost Europe, in particular (Watson et al., 2017). The best-suited crop for marginal agricultural areas is probably the pea (*Pisum sativum* L.).

The Pea has a long tradition in historical Nordic agriculture and has probably been cultivated in the region since the Bronze Age, although grain legumes seldom are preserved as archaeological remains (Regnell, 1998; Kirleis, 2019). The crop was most likely domesticated from wild *Pisum elatius* (Jing et al., 2010) and spread from the Fertile Crescent, among other agricultural founder crops, across the Mediterranean before it began its northward expansion (Colledge et al., 2005). Adaptation to novel light and temperature conditions must have been a necessity as pea cultivation gradually moved north. Historically and today, the pea is the most important protein crop in the Nordic countries (Osvald, 1959; Watson et al., 2017). At present, the pea is cultivated on 40 kha in Sweden (Statistics Sweden, 2020), 25 kha in Finland (Statistics Finland, 2020), and in Denmark on 3.8 kha for consumption and 7.4 kha for feed (Danmarks statistik, 2020). In Norway, the total legume cultivation only covered 3.9 kha in 2019 (Landbruksdirektoratet, 2020).

Cultivation is concentrated in the southern parts of the region, however, primarily because yield security is not sufficient in the region around and above the Arctic Circle with its colder climatic conditions. This problem could be solved by improving the existing germplasm through adaptation.

The gene bank at the Nordic Genetic Resource Center (NordGen) holds exceptionally rich resources of pea germplasm, not least of landraces gathered in the Nordic countries (Leino and Nygårds, 2008), as well as Nordic cultivars and breeding material. Molecular genetic analyses of this material show a great variability and high genetic differentiation among accessions from different geographical areas (Hagenblad et al., 2013; Leino et al., 2013; Solberg et al., 2015).

The pea has been cultivated in the Nordic countries under a range of climatic conditions with a growing season ranging from approximately 140–220 days. Thus, adaptive traits such as the time for flowering vary significantly in the material. Vanhala et al. (2016) studied a set of Swedish landraces under controlled greenhouse conditions and found a strong correlation between the days required for flowering and duration of the growth season at the site of origin. The pea is a typical facultative long-day plant and is therefore relatively insensitive to the variation in the length of day occurring in high altitude regions. The flowering time is dependent on the alleles in the *HIGH RESPONSE TO THE PHOTOPERIOD* (*HR*) gene, but also on other unknown genetic factors contributing to this trait. Several of the accessions that appear adapted to the Northern cultivation conditions, but are currently only cultivated in household gardening, have the potential for use in large-scale cultivation.

Adaptation and yield potential greatly depend on the flowering times and duration of growth. In turn, these traits vary among genotypes due to differences in their phenological response to temperature and photoperiods (Gottschalk, 1988; Wilson and Robson, 2006). The genetic control of this variation is well known (Paton, 1968; Berry and Aitken, 1979; Yan and Wallace, 1998; Weller and Ortega, 2015), but the phenotypic expression of responses to light and temperature is not well-defined. Knowledge about the responses of different genotypes to contrasting climates in terms of their phenology and yield components is important for the selection of the most appropriate cultivars and breeding materials for each environment. The information can also contribute to predicting the effects of climate change on agricultural production.

Most crop physiological studies so far have focused on responses to warm climates and heat stress. For example, Anwar et al. (2015) found dramatic effects on pea phenology and yield in a projected warmer climate in Australia. Huang et al. (2017) exposed a set of recombinant inbred lines (RILs) to heat stress through delayed sowing and found that flowering accelerated and yield decreased, but differed based on the genotype. Sadras et al. (2019) evaluated RILs segregating in phenology in different environments in Australia and found that flowering time had high heritability, whereas the later developmental stages were greatly affected by the environment. In contrast, there are fewer field trials that are performed at high latitudes, investigating the response of different genotypes to extreme day length, low temperatures, and a shorter growing season.

The major objective of this study was therefore to evaluate the responses of a wide range of genotypes in terms of phenology and yield to the specific climatic conditions of Arctic and sub-Arctic latitudes. Fifty accessions with a Nordic landrace or cultivar origin were cultivated in four contrasting environments, with two of the locations situated a long way north of the present-day cultivation area for peas. The phenology and yield parameters were recorded and analyzed in response to climatic variables.

## Materials and Methods

### Plant Materials

Fifty pea accessions from the collection at NordGen were selected for the trials (Table 1). The accessions were chosen based on the available documentation, such as place of origin and cultivation history. In particular, early flowering accessions with a short flowering time, primarily from the northern part of Finland, Norway, or Sweden, were picked. Sugar, shelling, and field pea types were obtained from both white flowering as well as color flowering *Pisum sativum* L. Furthermore, the accessions represented the different improvement groups, with two accessions classifying as breeding lines, 12 as cultivars and 36 as landrace material.

**Table 1:** Information about accessions in field trials in 2018 and 2019.

**Accession no**.	**Accession name**	**Improvement level (release year)**	**Seed type**	**Origin country**
7128	Norrøna	Cultivar(1958)	Sugar pea	NO
10778	Aslaug	Cultivar(1989)	Sugar pea	NO
11149	Jærert	Landrace	Shelling pea/Field pea	NO
11750	Sockerärt från Arvidsjaur	Landrace	Sugar pea	SE
13469	Stäme	Landrace	Field pea	SE
13784	Marma	Cultivar (1958)	Field pea	SE
14642	Lit	Landrace	Field pea	SE
17650	Sunna	Cultivar (1995)	Field pea	FI
17832	Farmor	Landrace	Shelling pea	SE
17833	Ögonsockerärt från Boaryd	Landrace	Sugar pea	SE
17837	Svartbjörsbyn	Landrace	Sugar pea	SE
17839	Vallagården	Landrace	Shelling pea	SE
17842	Edsås	Landrace	Shelling pea	SE
17855	Tant Erika	Landrace	Sugar pea	SE
17859	Solleröärt	Landrace	Field pea	SE
17863	Saxbo	Landrace	Shelling pea	SE
17865	Enviken	Landrace	Shelling pea	SE
17866	Biskopen 2	Landrace	Sugar pea	SE
17869	Kärrboda	Landrace	Field pea	SE
17873	Puggor från Ballingslöv-Glimåkra	Landrace	Field pea	SE
17882	Gästrikland	Landrace	Field pea	SE
18057	Martha	Landrace	Shelling pea	SE
18059	Avestaärt	Landrace	Shelling pea	SE
18680	Sumo	Cultivar (1995)	Sugar pea	DK
20011	Hedenäset	Landrace	Sugar pea	SE
20012	Delikatess	Cultivar (1905)	Sugar pea	SE
20043	Lom	Landrace	Field pea	NO
20121	Marie	Landrace	Shelling pea	NO
20201	Grötom	Landrace	Shelling pea	SE
20205	Gaperhult	Landrace	Shelling pea	SE
21659	Ringeriksert	Landrace	Field pea	NO
21951	Signal	Cultivar (1995)	Sugar pea	DK
22830	Raber	Landrace	Field pea	SE
22832	Gendalens ärter	Landrace	Shelling pea	SE
23819	Tidlig lav	Cultivar	Sugar pea	NO
24333	Bjurholms blåärt	Landrace	Shelling pea	SE
24334	Bjurholms gråärt	Landrace	Field pea	SE
24335	Bjurholms småärt	Landrace	Field pea	SE
24765	Karls høje ært	Landrace	Shelling pea	DK
100930	Klosterärt	Cultivar (1945)	Field pea	SE
101109	Strål	Cultivar (1955)	Field pea	SE
102222	WBH2222	Landrace	Field pea	SE
103076	Jämtländsk gråärt	Landrace	Field pea	SE
103488	WBH3488	Landrace	Field pea	SE
103491	Hja 10953	Breeding material	Field pea	FI
103496	Hja 51229	Breeding material	Field pea	FI
103523	WBH3523	Landrace	Field pea	SE
103549	Elin	Landrace	Sugar pea	SE
103826	Simo	Cultivar(1973)	Field pea	SE
103853	Inkilän herne	Landrace	Field pea	FI
Reference	Ingrid	Cultivar(2010)	Field pea	SE
Reference	Karita	Cultivar(1995)	Field pea	SE

*DK, Denmark; FI, Finland; NO, Norway; SE, Sweden*.

Many of the accessions in the study were regenerated in Taastrup, Denmark, in 2017, and the new seed material could be used in this study. For the remaining 17 accessions, seeds of good quality and in good amount were already available. Depending on the estimated germination percentages for the individual accessions, sufficient seed quantities were aliquoted by NordGen's seed laboratory. Seeds for two replicated trials performed in 2018 and 2019 were prepared and stored in NordGen's freezers until the time of sowing. The accessions for the two trial years were identical, except for two reference cultivars, “Ingrid” and “Karita,” which were included in 2019. Seeds for these two accessions were received from Boreal Plant Breeding Ltd. in Finland.

### Field Trials

**Figure 1:**
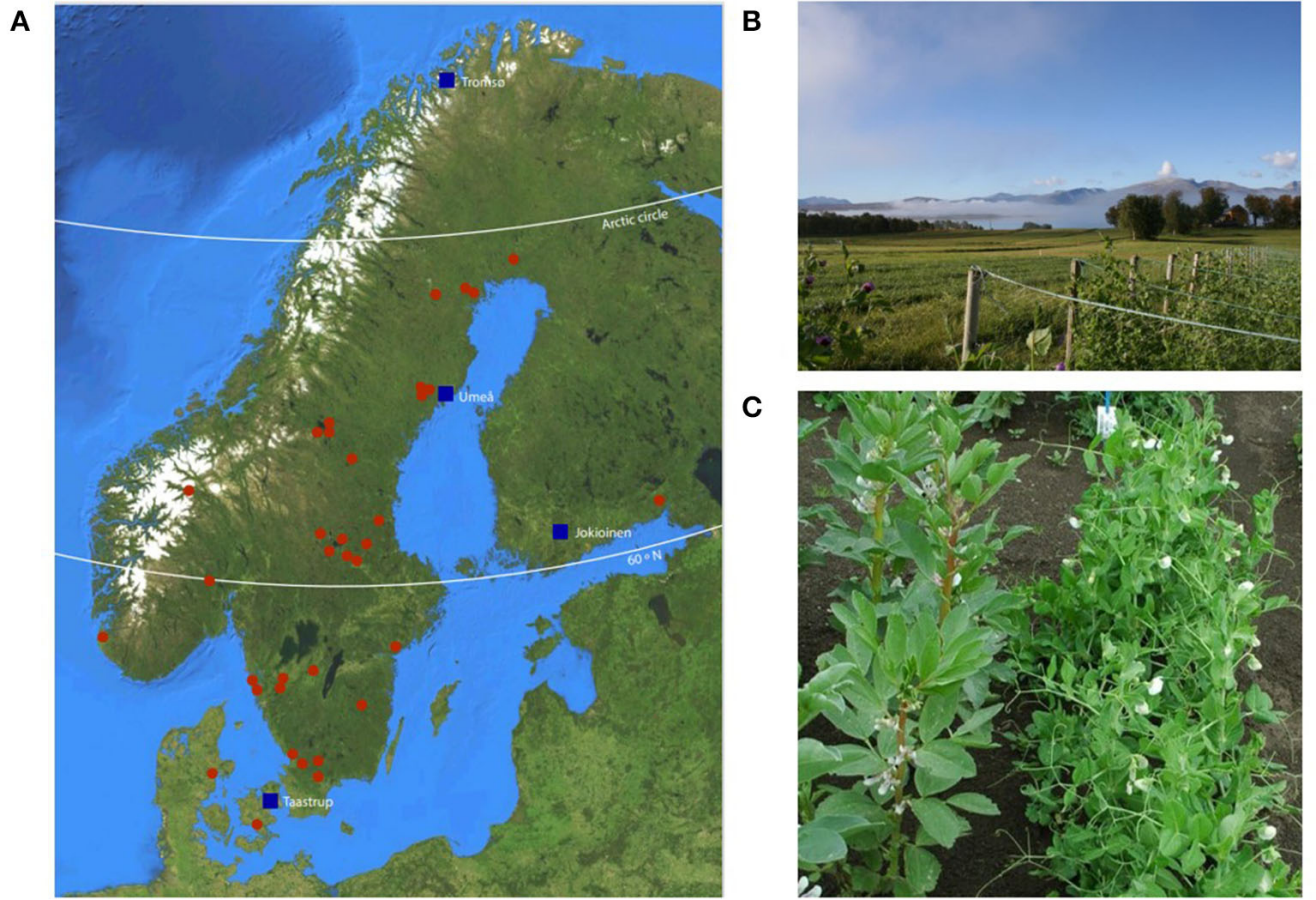
**(A)** The map of trial sites (blue squares) and landrace accessions with identified locations of origin (red dots). **(B)** The Tromsø trial site (Norway). Photo by Ulrika Carlson-Nilsson, NordGen, Sweden. **(C)** The Jokioinen trial site (Finland) where fava beans were used as climbing support. Photograph by Sanna Kulmala, Natural Resources Institute, Finland.

Four contrasting growing locations of latitudes ranging from 55° to 69°N were selected. Seeds were distributed to the trial sites in Denmark (Taastrup, 55°40′ N; 12°18′ E), Finland (Jokioinen, 60°48′ N; 23°29′ E), Sweden (Umeå, 63°49′ N; 20°11′ E), and Norway (Tromsø, 69°39′ N; 18°54′ E) (Figure 1A).

The trials at all four locations had an identical layout with four blocks, each following a randomized order. In 2019, all the trials followed the same layout, but with a different randomized order within the blocks compared to that in 2018.

At all sites, the number of seeds of each accession were sown according to the estimated germination percentages, and plants were counted after emergence, aiming at 20 plants per accession and block. If the number of established plants per plot greatly exceeded 20, the surplus was removed. Each plot for the 20 plants per accession was a two-meter row (in Jokioinen, 2 × 1-m rows), with an average of 10 cm between plants. However, the total number of plots in a row varied between sites.

Date of sowing for the sites varied between 3 May (Taastrup, Denmark) and 12 June (Tromsø, Norway) in 2018, whereas in 2019 sowing took place between 2 May (Taastrup) and 24 June (Umeå, Sweden) (Table 2).

**Table 2:** The sowing dates.

**Trial site**	**Sowing date (2018)**	**Sowing date (2019)**
Taastrup, Denmark	3 May	2 May
Jokioinen, Finland	24 May	21 May
Umeå, Sweden	25–29 May	20–24 June
Tromsø, Norway	12 June	5 June

The model of field establishment and cultivation practices varied at the different sites. At Taastrup and Tromsø, the seeds were hand sown, at Jokioinen they were sown with a plot seeder directly into the field in both years, while in Umeå in 2018 they were sown into small pots at room temperature between 25 and 29 May, placed outdoors and covered with a non-woven fiber on 31 May to acclimatize them, and then transplanted to the field between 19 and 21 June. In 2019, seeds were sown directly into the field at this site as well.

In Denmark, Sweden, and Norway, accessions were cultivated with poles and nets for climbing support in both years (Figure 1B). In Finland, fava beans were used for support instead (Figure 1C). Drip hoses were used for automatic irrigation in Denmark, while at the other sites in both years manual irrigation was carried out when needed.

The trial in Umeå, Sweden, was fertilized with NPK 11-5-18, 450 kg/ha on 15 June in 2018 and with NPK 11-5-18 545 kg/ha, on 12 June in 2019. No other treatments were performed.

The trial in Tromsø, Norway, was fertilized with 200 kg/ha potassium sulfate (41% K, 18% S) as a strip application at the time of sowing in both years. No pesticides were used during the cultivation period.

The trial in Jokioinen, Finland, prior to sowing, was fertilized with NPK 23-3-8, 175 kg/ha in both years. The weeds were controlled with two applications: Fenix (active ingredient aclonifen 600 g/l, Bayer Crop Science) 3 l/ha 5 days after sowing, and Senkor WG (active ingredient metribuzin 700 g/kg, Bayer Crop Science) 0.1 kg/ha after plant stand establishment.

In Taastrup, Denmark the trial was fertilized with 600 kg/ha NPKS 0-4-21-6 in both years in mid-April. On 15 May Karate 2.5 WG (active ingredient lambda-cyhlothrin 2.5 g/l, Syngenta Nordics A/S) 0.2 kg/ha and on 1 June 2018 Mavrik 2F (active ingredient tau-fluvalinate 240 g/l, Adama Northern Europe B.V.) 0.2 l/ha were used to treat against pea weevils. On 29 June, Mavrik 2F 0.1 l/ha was also used against pea moth. In 2019, treatments were performed on three occasions: Mavrik Vita (active ingredient tau-fluvalinate 240 g/l, Adama Northern Europe B.V.) 0.2 l/ha against pea weevil on 28 May, Ferrex (active ingredient iron (III) orthophosphate 2.5% (25 g/kg), DLG Denmark) 6 kg/ha against snails and slugs on 9 June, and Mavrik Vita 0.2 l/ha against aphids on 28 June.

Rhizobium was not applied at any of the sites in either year.

### Field Evaluations

In both years, observations and evaluations of traits were performed at all sites during the growing season. As far as possible, the same person at the respective site performed all evaluations of the same trait throughout each season. The focus was on characters of importance for successful cultivation in more northern regions and a number of more common traits were also evaluated (Table 3).

**Table 3:** The traits evaluated.

**Trait**	**Sub-trait**	**Description**
Stem height/length		Measured on five randomly chosen plants at time of full flowering (cm)
Flowering time	Start of flowering	Number of days after sowing when 10% of the plants have flowers
	Full flowering	Number of days after sowing when 90% of the plants have flowers
	End of flowering	Number of days after sowing when 90% of the plants have no flowers
Maturation time	Green maturation	Number of days after sowing when 25% of the plants have pods that are swollen, and peas fill the pods
	First maturation	Number of days after sowing when 10% of the plants have mature pods (dry pods with dry and hard peas)
	Full maturation	Number of days until 90% of the plants have mature pods
Yield	Fresh biomass	Weight (gram) per plant, whole plant^*^
	Dry biomass	Weight (gram) per plant, whole plant after drying at a maximum of 40°C, until dry
	Seed	Weight (gram) per plant
	TGW	Weight (gram) of 1,000 seeds

* *Harvest was performed at full maturation or, if not reached, at the end of the growing season*.

### Climate Conditions

**Figure 2:**
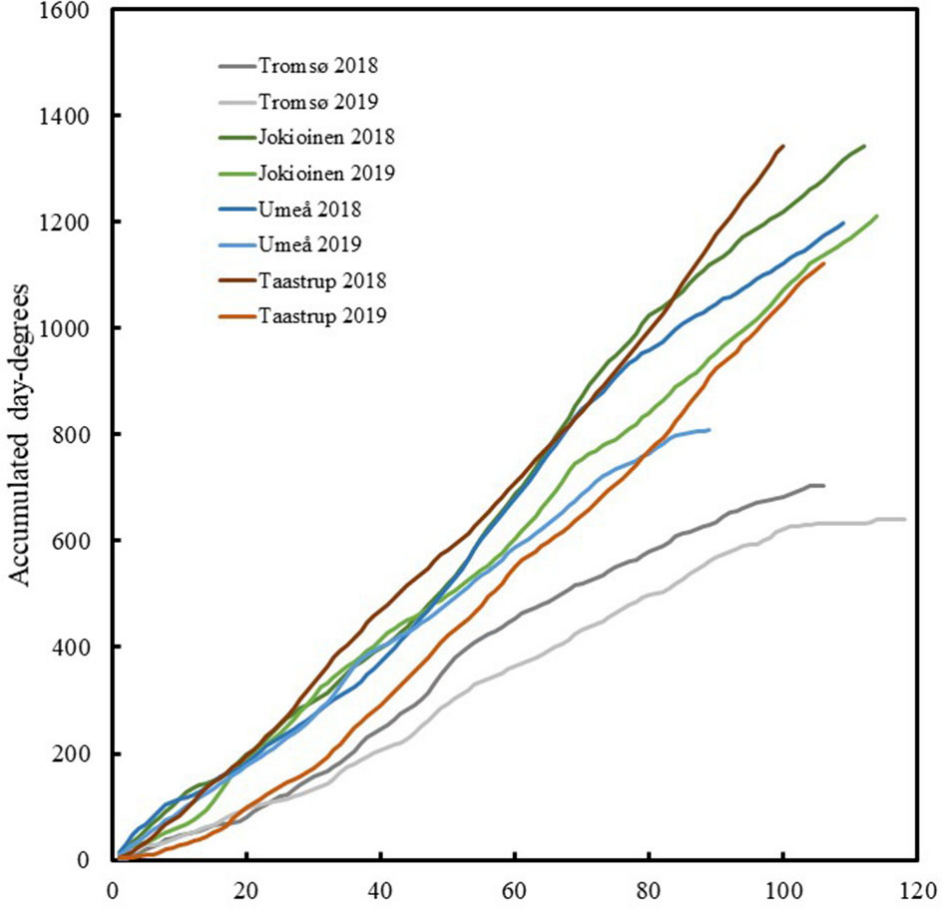
The accumulated day degrees from date of sowing for different sites and years.

Meteorological data for daily values of precipitation and hourly values of air temperature and global radiation were downloaded from meteorological stations at the locations for the field trials or in their proximity (0–3 km). Meteorological data from 2018 to 2019 at Taastrup, Denmark were downloaded from Svane et al. (2020). Data at Umeå, Sweden were downloaded from SMHI (SMHI, 2020). Data from the Holt agricultural research station in Tromsø, Norway were downloaded from Norsk Landbruksmeteorologisk Tjeneste (Norsk Landbruksmeteorologisk Tjeneste, 2020). Data from Jokioinen, Finland were provided by the Finnish Meteorological Institute (Finnish Meteoroligical Institute, 2020). The average days for the developmental stages (first flowering, full flowering, green maturation, and full maturation) for each accession were related to the accumulated day degrees per test site and year, with the base temperature set at 5°C. The temperatures at all sites in 2018 were consistently higher than those in 2019 (Figure 2, Table 4).

**Table 4:** The average temperature, total precipitation, total global radiation, average daily photosynthetic light period (PAR), and the average photoperiod during the field trials in 2018 and 2019.

**Location, year**	**Temp**.	**Total precip**.	**Total radiation**	**PAR**	**Photo-period**	**Exp. period**
	**(^**°**^C)**	**(mm)** ^*^	**(MJ/m^**2**^)**	**(h)**	**(h)**	**(days)**
Tromsø 2018	11.6	308.6	1173.2	13.9	19.7	106
Tromsø 2019	10.4	286.5	1489.3	14.6	19.6	118
Umeå 2018	16.0	162.6	2087.6	16.0	18.3	109
Umeå 2019	14.1	175.8	1462.4	14.9	17.3	89
Jokioinen 2018	17.0	122.1	2019.9	15.4	17.3	112
Jokioinen 2019	15.6	223	2108.2	15.0	17.3	114
Taastrup 2018	18.4	62.6	2329.6	15.7	16.8	100
Taastrup 2019	15.6	211.3	2090.5	15.2	16.7	106

*The experimental period is the number of days from sowing to the end of the trial, and averages and totals are based on these numbers of days for the respective location*.

* *Drip hoses were used in Taastrup in both years and manual irrigation was carried out at other sites when needed*.

The total global radiation during the field trials was highest at the southernmost site in Taastrup, Denmark and lowest at the northernmost site in Tromsø, Norway in 2018, and in Umeå, Sweden in 2019 when seeds were sown in late June (Table 4). Conversely, precipitation was lowest in Taastrup, Denmark in 2018 at 63 mm and highest in Tromsø at 309 and 287 mm in 2018 and 2019, respectively. The photoperiod for each site and year was obtained on a daily basis using the NOAA Solar calculator (2020), and ranged from an average of 16.7 h in Taastrup to 19.7 h in Tromsø. The average estimates of photosynthetic light periods (PAR) were also calculated based on the daily duration of global radiation above 50 W/m on a daily basis, assuming a linear change per measured 24-h time points per day. Even though the photoperiod was 24 h in mid-summer in Tromsø and the maximum PAR duration of 22.5 h was observed on a daily basis, cloudiness and rapidly decreasing day lengths in autumn compared with the other sites resulted in the lowest average PAR, on a daily basis, for Tromsø in both years. The PAR at the other locations/years ranged between 14.9 and 16.0 h (Table 4).

### The Harvest

The harvest took place at the various sites when plants reached full maturation (identified as dry pods with dry and hard peas) or, in case of the accessions being too late, before the end of the growing season. At harvest, plants were cut at ground level and each accession within each block was put into net bags and dried. The weight of the total biomass was measured before and after drying.

After drying, threshing was performed by hand as in 2017 it had been observed that seeds were too heavily damaged if they were threshed by machine. Threshing was either performed at each site or at NordGen (harvests from Umeå, Sweden and Taastrup, Denmark in 2018, and from Taastrup in 2019).

In both years, where a sufficient harvest had been obtained, seed yield and thousand grain weight (TGW) of each accession and block were measured.

A sample of 50 grams was then taken and sent for protein analysis. The protein concentration was measured by Boreal Plant Breeding Ltd., in Jokioinen, Finland with a near-infrared (NIR, FOSS InfraXact) analyser with a wavelength of 570–1,850 nm. Samples were sieve-milled using Falling Number Laboratory Mill 3,100 with a 0.8 mm sieve. In 2018, the mature peas were available for protein analysis from Taastrup, Jokioinen and Umeå, and in 2019 from Taastrup and Jokioinen.

### Data Analysis

The mean days from sowing to the observed first flowering, full flowering, green maturation, first maturation, and full maturation were analyzed using Minitab®19 (v. 19.2), by GLM ANOVA across cultivars, with location and year as fixed variables. Mean days for cultivars (across location and year) were analyzed by the one-way ANOVA.

Mean estimations for variables related to yield, yield components, and protein were calculated by SAS software's MIXED procedure using version 9.4 (SAS.inc, 2020). The data were analyzed using linear mixed models. The Square root, logarithm, or *arc sin* square-root transformation was done prior to analysis to normalize random variances. However, all the estimates were transformed back to the original scale for presentation purposes. Accessions were classified into three types: sugar, shelling, and field pea. The trial variable identified the experiment by location and year. The type of accession and accession nested within the type of accession were fixed factors in the linear mixed model. Random effects included the trial (location-by-year combination), blocks nested within the trial, and the accessions nested within the type of accession-by-trial interaction effect. Correlation analysis was carried out computing Pearson correlation coefficients by the SAS Corr procedure using version 9.4 (SAS.inc, 2020).

## Results

The study included a collection of 50 pea accessions comprising 11 cultivars (released from 1905 to 1995), two breeding lines and 37 landraces (Table 1). The landraces all have a long history of cultivation in Nordic countries. The accessions were evaluated for performance at four locations from latitude 55° to 69°N above the polar circle and from longitude 12° to 23°E. The field trials were carried out for 2 years, and at two locations, Taastrup (55°N), Denmark and Jokioinen (60°N), Finland; all accessions could be harvested at full maturation, whereas those in the sub-Arctic regions gave more variable results regarding flowering, green harvest, and maturation.

### Phenology and Thermal Requirements

**Figure 3:**
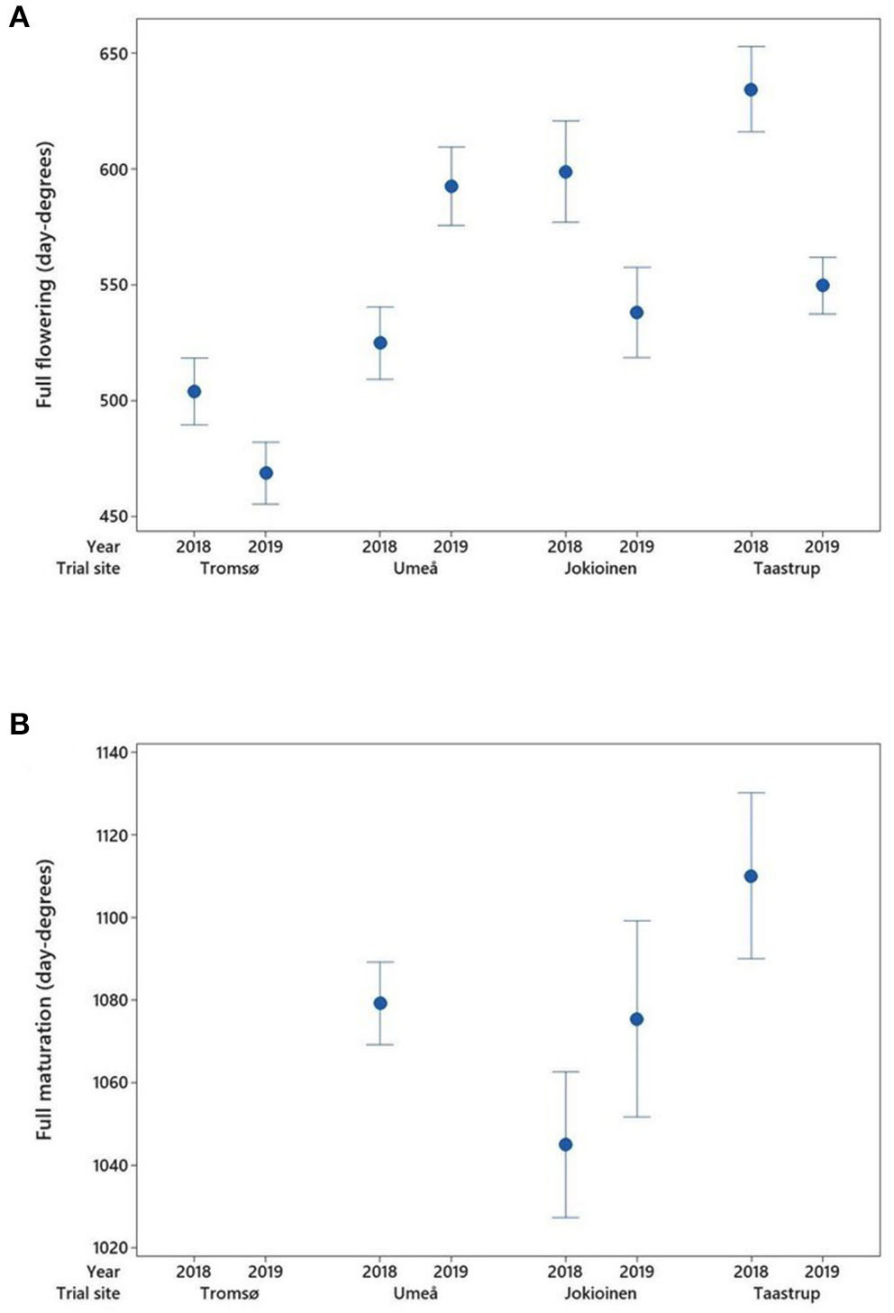
Average day-degree sums at different sites and years for the development of **(A)** full flowering and **(B)** full maturation in (*n* = 49–52) accessions, with error bars indicating a 95% confidence interval. There was no development of mature pods in Tromsø in either year or in Umeå in 2019. The data for full maturation were not recorded and are therefore missing at Taastrup for the year 2019.

Since the field experiments were performed in different environmental conditions, the phenological development stages are expressed in day degrees (with the base temperature at 5°C) rather than number of days. The thermal sums required for the start of flowering and the full flowering (Figure 3A) differed between sites and years (*P* < 0.001). In both years of the trial, the start of flowering and the full flowering in the northern most location of Tromsø, Norway were consistently reached in lower day-degree sums than other locations. When using the number of days for comparison, however, the northernmost site took longer to reach these stages (mean 67 days in 2018 and 75 days in 2019) compared with the other sites (mean range 49–59 days). On average across accessions and years, the difference from the lowest to the highest latitude for the full flowering was in the magnitude of 100 day degrees. For all locations except Umeå, Sweden the flowering stages were reached at a higher number of day degrees in 2018 than in 2019, although it should be noted that sowing in Umeå in 2019 was undertaken several weeks later than in the previous year.

In the two lowest latitudinal sites of Taastrup, Denmark and Jokioinen, Finland, full maturation was reached for all accessions in both years (Figure 3B). In Umeå, Sweden, all accessions reached full maturation in 2018, but none did so in 2019. In the northernmost location of Tromsø, Norway, no accessions reached full maturation in either year, with just 21 accessions reaching the “green maturation” stage in 2018 and 35 in 2019. There was no significant difference between location/years for full maturation, with mean day degrees in the range of 1,045–1,110.

### The Earliest Accessions

**Figure 4:**
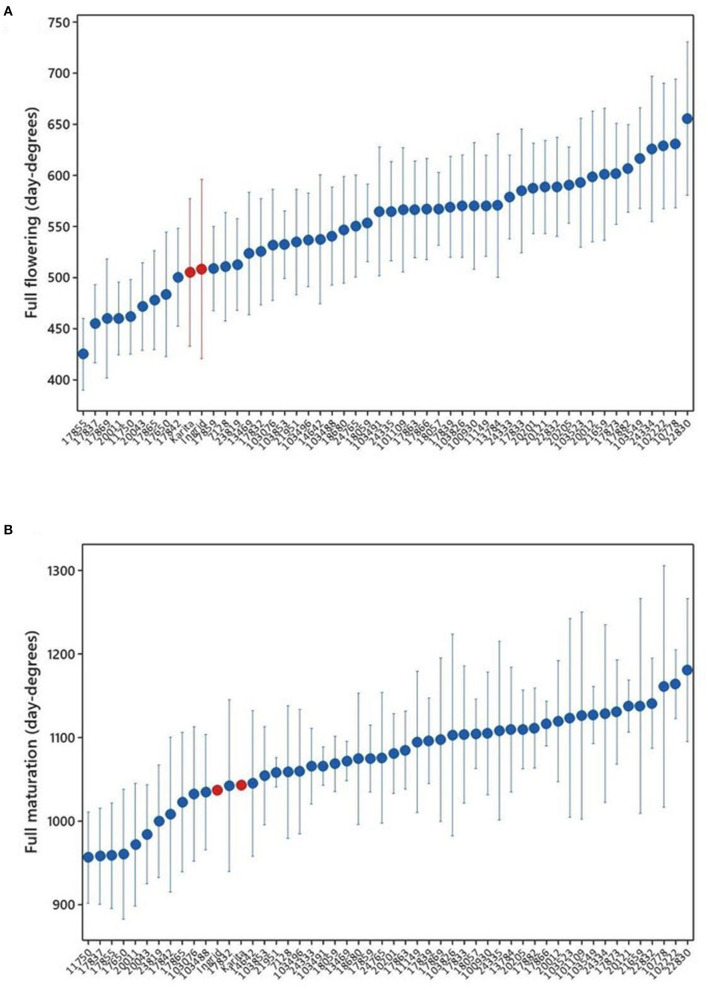
Average day-degree sums for the development of **(A)** full flowering, sample size *n* = 7–8 in all accessions, except reference cultivars “Karita” and “Ingrid” (in red color), which were only tested in 2019 (*n* = 4), and **(B)** full maturation of pods in accessions grown at all sites and in both years. The development of fully mature pods was observed at (*n* = 4–5) location x years, except for “Karita” and “Ingrid” in 2019, where there were data only from Jokioinen. There was no development of mature pods in Tromsø in either year and in Umeå in 2019. The data for full maturation were not recorded and are therefore missing at Taastrup for the year 2019.

There was a significant difference in accumulated day degrees between the cultivars for all the observed developmental stages: first flowering (*P* < 0.001), full flowering (*P* < 0.001), green maturation (*P* = 0.008), first maturation (*P* < 0.001), and full maturation (*P* < 0.001). The full flowering means ranged from the lowest at 425 day degrees for the sugar pea landrace “Tant Erika” (17855) to the highest at 656 day degrees for the landrace “Raber” (22830) (Figure 4A). There were nine cultivars with shorter means than the reference cultivars “Karita” and “Ingrid,” mostly sugar pea varieties, several with northern origins in Sweden. The full flowering, expressed in the number of days, was reached within the range of 47–68 days. The order of means for the full maturation of pods was similar to those for flowering, with the sugar pea landrace “Sockerärt från Arvidsjaur” (11750) originating in northern Sweden at the lowest of 956 day degrees and again the landrace “Raber” (22830) was highest at 1,180 day degrees (Figure 4B). In the sites where full maturation was reached, the number of days required for this stage spanned a mean range of 82–99 days.

The accessions that reached full flowering earlier than reference cultivars in terms of day degrees and across all locations and years were the sugar peas “Tant Erika” (17855), “Svartbjörsbyn” (17837), “Hedenäset” (20011), and “Sockerärt från Arvidsjaur” (11750); the shelling peas “Enviken” (17865) and “Edsås” (17842); and the field peas “Lom” (20043), “Kärrboda” (17869), and “Sunna” (17650) (Figure 4A). All are landraces, except for “Sunna,” which is a cultivar from Finland. Although their individual order varied somewhat between locations, these accessions were consistently among the earliest to reach the full flowering stage at all locations.

Since two of the trial locations are at latitudes well above the areas for commercial pea production, it was not expected that all accessions would reach full maturation. To be able to give a tentative ranking of the accessions after the full flowering stage in these locations, the phenological stage “green maturation” was introduced. The “green maturation” stage corresponds to the field pea BBCH growth stage 79 (green ripe). In the northernmost location of Tromsø, Norway the annual average day-degree sum (based on the 30-year normal, 1961–1990) was only 612. All of the earliest accessions at the full flowering stage were among those that also reached the “green maturation” stage. In Tromsø in 2018, 21 accessions reached “green maturation” in day-degree sums ranging from 689 to 702, while the numbers for 2019 were 35 accessions in day-degree sums ranging from 562 to 640. The earliest accession to reach “green maturation” in Tromsø in both years was the field pea landrace “Lom” (20043), originating from a mountainous/high-altitude location in Norway, which has a short growing season. This accession also differed from all other accessions by being exceptionally short and compact in growth, with an average height below 20 cm in all locations.

### Yield Components and Seed Traits

In Tromsø, Norway, accessions failed to reach dry seed maturation in both years, and this was also the case in Umeå, Sweden in 2019 where sowing dates were delayed compared to 2018. The seed yield (g/plant) varied greatly across locations and years. The average seed yield across the accessions was 13.3 g/plant in Taastrup, Denmark, 2.0 g/plant in Jokioinen, Finland, and 7.8 g/plant in Umeå, Sweden in 2018. The low yield in Jokioinen was due to low precipitation during the sowing period and drought in the early growing season. This resulted in poor and uneven seedling emergence and weak growth. In 2019, the average seed yield was 9.5 and 17.1 g/plant in Jokioinen and Taastrup, respectively.

**Figure 5:**
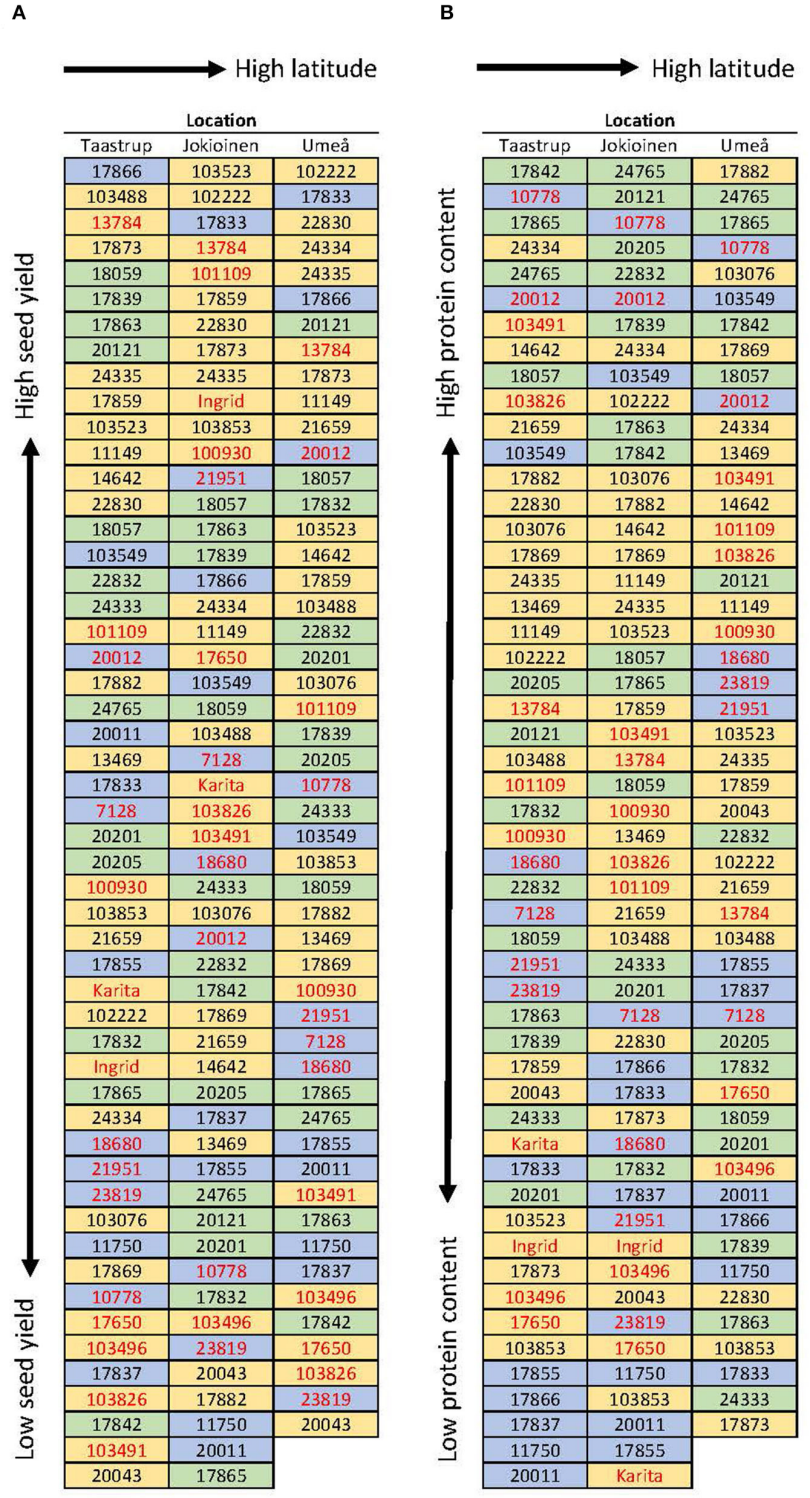
Ranking of accessions regarding **(A)** seed yield gram per plant and **(B)** protein percent. The values are based on the average of 2 years (2018 and 2019) except for Umeå, which is based on values obtained for the year 2018 alone. The blue background represents the sugar pea, the green represents the shelling pea, and the yellow background represents the field pea. Cultivars and breeding lines are shown in red, the rest are landraces.

The modern cultivars, “Ingrid” and “Karita,” were included in 2019 as reference genotypes. In terms of seed yield across locations, these performed well—“Ingrid” 9.3 g/plant and “Karita” 8.5 g/plant—but were not among the highest-yielding genotypes at any location (Figure 5A). The accessions “Biskopen 2” (17866, sugar pea), “Marma” (13784, field pea), “WBH3523” (103523, field pea), and “Puggor från Ballingslöv-Glimåkra” (17873, field pea) were among the top-yielding (13.2–14.0 g/plant) genotypes across locations and years, performing well at all sites (Figure 5A). These high-yielding accessions were all tall-stem types, with the average stem height ranging from 98 cm to 132 cm compared with 80 cm and 60 cm for “Ingrid” and “Karita,” respectively. Many sugar pea and shelling pea accessions produced dry seed harvests in the same range as the accessions aiming for dry seed production. Landraces and cultivars were not separated for the purpose of ranking the seed yield at any location (Figure 5A).

The accessions “Bjurholms gråärt” (24334, field pea), “Enviken” (17865, shelling pea), “Aslaug” (10778, sugar pea), and “Delikatess” (20012, sugar pea) had the highest protein concentration, above 27%, across locations and years, whereas in reference genotypes “Ingrid” and “Karita” (both field peas) protein was 22.2 and 21.4%, respectively (Figure 5B). In terms of the protein yield (g protein/plant), the same four genotypes producing the highest seed yield were also the highest protein yield producers (“Biskopen 2” 2.9 g protein/plant, “Marma” 3.0 g protein/plant, “WBH3523” 3.1 g protein/plant and “Puggor från Ballingslöv-Glimåkra” 3.4 g protein/plant). “Ingrid” and “Karita” produced 2.0 and 1.8 g protein/plant, respectively. No distinction was found between landraces and cultivars in terms of the protein content and many sugar peas and shelling pea accessions ranked at the top.

**Figure 6:**
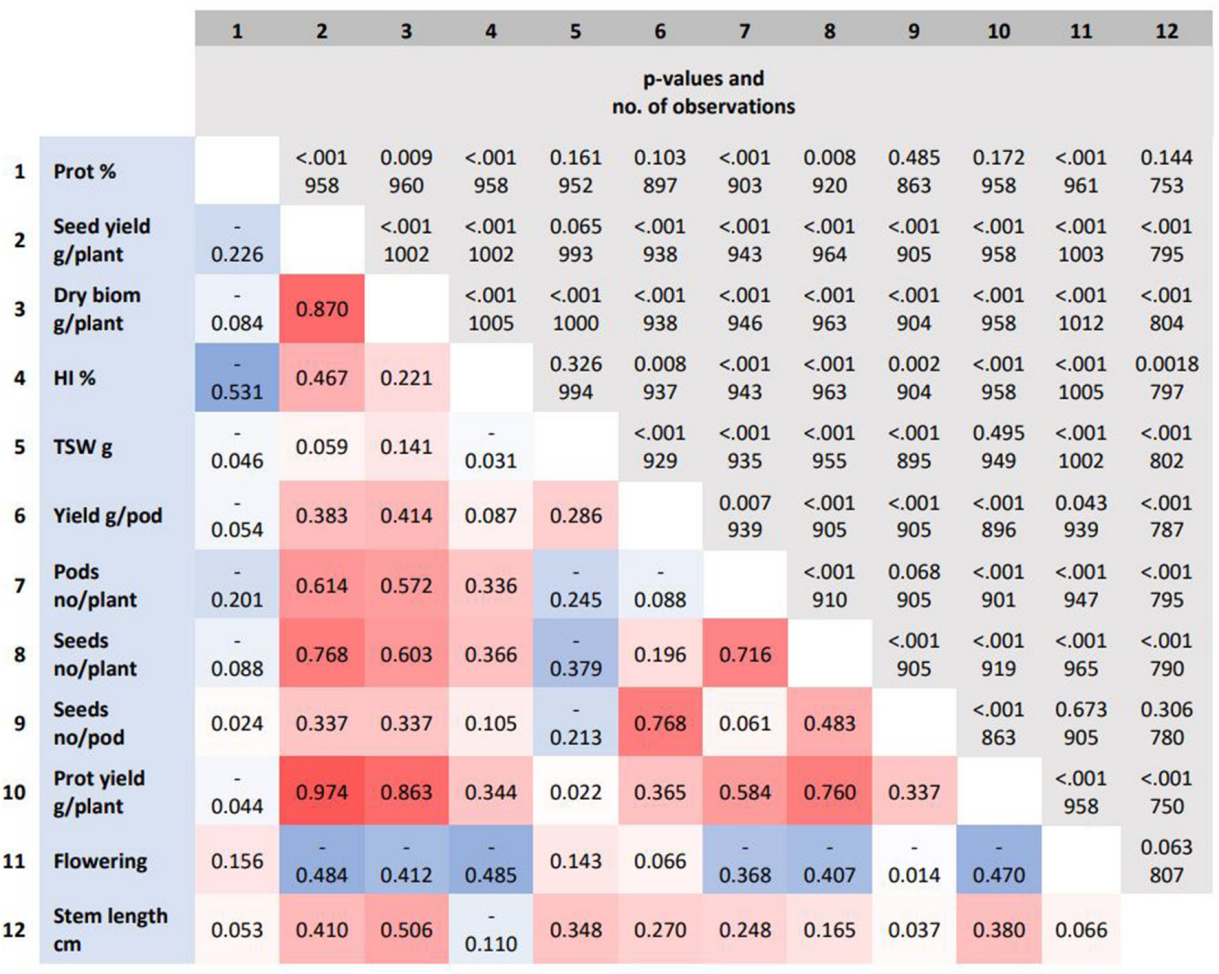
The Pearson correlation coefficients for yield and protein-related traits are represented by the colored background, while *p*-values and the number of observations are represented by the gray background. Flowering indicates first flowering. prot, protein; biom, biomass; HI, harvest index; TSW, thousand seed weight.

Despite variation in seed yield, the connection between seed yield with yield-associated traits was fairly similar across locations. The seed yield (g/plant) was more strongly associated with traits related to seed number determination than seed weight. The seed yield (g/plant) correlated more positively with the seed number per plant, the aboveground dry biomass (g/plant) and, to a lesser extent, with the harvest index (HI, %) and the stem length (cm). The seed yield (g/plant) correlated negatively with the start of flowering period. The protein concentration (%) correlated slightly negatively with seed yield (g/plant) and much more negatively with HI (%). The protein yield (g protein/plant) correlated more positively with the aboveground dry biomass (g/plant), the seed yield (g/plant), and traits related to the seed number, but not with protein concentration (%) (Figure 6).

## Discussion

### Phenology and Thermal Requirements

A major limitation to reaching full maturation in northernmost locations is low temperatures in spring and early summer, leading to delayed sowing times and slow germination. The main hindrance to crop production in northern growing conditions is the short duration of a favorable growing period. Despite acceleration in the development rate due to the long photoperiod at high latitudes, crops run the risk of failing to mature and/or harvest due to unfavorable weather conditions during the harvesting period (Peltonen-Sainio et al., 2014). Temperatures fall more rapidly at high latitudes in autumn due to lower solar elevations and increased shortening of day lengths in comparison with those further south (Nilsen, 1985). In addition, at high latitudes, there are also greater risks of late spring frosts as well as early autumn frosts. The requirement for up to 1,000 day degrees for full maturation in peas in Scandinavian summer conditions (Figures 3, 4) thus limits the possibility of cultivation above the Arctic Circle to early-flowering varieties of green maturing sugar peas. In the present tested material, historic varieties from areas near the Arctic Circle, particularly from Sweden, and one variety from a mountainous part of Norway stand out as possible candidates for production at high latitudes. Vanhala et al. (2016) found a strong correlation between the length of growing season at the location of origin and the flowering time in Swedish pea landraces. This and the direct importance of early flowering in these regions are confirmed by this study.

The present data show that a lower accumulated day-degree sum is required for flowering at the two northernmost field sites above the Arctic Circle in Tromsø, Norway and near it in Umeå, Sweden (Figure 3). This observation indicated that longer photoperiod and/or longer daily PARs in June/July can reduce the temperature requirement for flowering at high latitudes. A similar effect has been observed for the development of floral heads in broccoli grown across a wide latitudinal range from Tromsø to central Europe (Johansen et al., 2017). However, while the 24-h photoperiod is well above the threshold of 12–13 h for flowering in peas (Iannucci et al., 2008), flowering was recorded on average 15–20 days later in Tromsø than at the other sites, suggesting that average temperatures, below 14.4°C, observed at this site are too low. Sensitivity to a long photoperiod in peas varies between cultivars depending on temperature, with especially early varieties becoming insensitive at low temperatures (Berry and Aitken, 1979). However, in 2018, the observed difference between years in the required temperature sum accumulation to reach certain developmental stages, such as the start of flowering period, indicated that pea genotypes adapted to the northern growing conditions could not fully utilize the higher temperature for their development. Extremely high temperatures and dry conditions have been shown to shorten the vegetative growth and flowering period in field peas in Australia and southern Canada (Bueckert et al., 2015; Sadras et al., 2019). The threshold temperature for this negative impact on flowering is close to or above 28°C. In this study, the threshold temperature was exceeded at the southernmost location of Taastrup, Denmark in 2018, and accordingly a slightly shorter time (6 days) for full flowering was observed, compared with the cooler year of 2019.

### Yield and Yield Parameters

The yield of mature pea appears to depend more on the number of seeds than on the thousand seed weight (TSW) (Figure 6). This result is in accordance with earlier studies on pea (Hovinen, 1988; Sadras et al., 2019; Klein et al., 2020). The dominance of seed number over seed weight is a typical phenomenon in other seed-producing crops as well, such as cereals (Peltonen-Sainio et al., 2007; Rajala et al., 2017). In cereals, plant breeding has shortened the stem length and altered aboveground dry matter allocation from vegetative plant organs to generative ones, while no marked changes in total aboveground biomass have occurred (Bingham et al., 2012; Rajala et al., 2017). Also in peas, plant breeding has changed the aboveground architecture by incorporating *afila* and dwarf genes into pea cultivars (Hovinen, 1988; Hofer and Ellis, 1998; Reid and Ross, 2011). Leafless and semi-leafless types combined with a shorter stem type result in lower aboveground biomass, a slightly lower seed yield, but a higher proportion of total above ground biomass were seeds, i.e., increased harvest index (Snoad, 1981). In this study, only three out of 50 accessions were *afila* types (Supplementary Material). When considering the (feed) value of a crop, an equally important trait as seed yield is the protein yield (kg protein/ha) produced by the crop. In this study, protein yield was strongly associated with seed yield, but not with the seed protein concentration (Figure 6). This is in accordance with an earlier study by Hovinen (1988).

In this study, stem length and the late start in flowering were associated with a higher seed yield (Figure 6). However, accessions with these traits have a higher risk of never reaching maturation in locations with insufficient day degrees. Some of the landraces and older cultivars showed a superior yielding capacity compared to the reference cultivars (Figure 5A). However, the agronomic traits of landraces and old cultivars contrast with modern cultivars in several aspects. Non-uniformity, late maturation, and tall stems inducing lodging susceptibility and complications in combine harvesting are particularly undesirable traits that restrict the large-scale cultivation of landraces and old cultivars in modern agriculture. It should also be noted that these field trials were conducted using supporting nets or support crops. In a free-standing population, tall-stem types probably perform worse. Nonetheless, these landraces and old cultivars can provide an alternative source of genetic material, both for breeding and direct cultivation, to broaden and diversify pea cultivation in Nordic countries. A high yield of mature pods and protein content in types (sugar pea, shelling pea) primarily intended for the green harvest of pods or immature seeds was also observed (Figure 5B). The gene pools of garden-type peas used for fresh harvest and field-type peas used for the harvest of dry seed overlap (Baranger et al., 2004; Hagenblad et al., 2013), which partly explains this finding. In addition, the protein content as well as composition (albumin, legumin and vicilin) and starch content are known to vary between smooth and wrinkled seeds, depending on the genes at the *rugosus* loci (Wang and Hedley, 1991; Perez et al., 1993). Both seed types were represented within the groups of garden peas and field peas in this study (Supplementary Material). The high yield of dry seed in many garden types indicates that these may contain genetic variability that can contribute to yield in dry seed cultivars.

### Cultivation of Peas in the Arctic Region

The cultivation of peas in the Arctic region is challenging. At the northernmost location of the field trial, no accession reached full maturation in either year. It should nevertheless be noted that green harvest of peas as a fresh vegetable was still possible at the northernmost location, suggesting that cultivation of garden-type peas for green harvest is the best alternative in most extreme conditions. Historically, garden peas have indeed been produced in these northern areas. For example, during the Second World War, several hundred farms or market gardens commercially produced garden peas in the two northernmost counties of Sweden, Västerbotten and Norrbotten (Statistiska centralbyrån, 1944). Many of the best performing accessions in Tromsø, Norway and Umeå, Sweden were sugar pea landraces gathered from northern locations. These could be a starting material for adapting the pea to the Arctic.

Whatever the end result, pea cultivars suitable for cultivation in the Arctic have special trait requirements. Ideotypes for pea under different conditions have previously been investigated. In the French PeaMUST project, the focus was on plant architecture and resistance traits to avoid stress (Burstin et al., 2018). Annicchiarico and Filippi (2007) identified traits suitable for organic production in Northern Italy, such as weed competition, while Castel et al. (2017) investigated the winter hardiness traits important in French winter peas in response to climate change. When it comes to pea ideotypes suitable for Arctic conditions, phenology must be prioritized when identifying ideotypes. The most useful investigation done so far was by Hovinen (1988) in a study of field peas in Finland. Besides the importance of lodging resistance traits, which were not studied here, Hovinen found that a growth period of 91–101 days and a flowering period of 19–28 days were optimal. In the more northerly locations tested here, where that number of days are not available, rapidly developing cultivars would be more suitable. In the Arctic, the agricultural practices and the time of sowing for pea cultivation will be extremely important in using available light and temperature as efficiently as possible.

### Perspectives of Climate Change

All climate change scenarios predict significant changes that have future implications in agriculture in the Arctic and the sub-Arctic regions. A study by Uleberg et al. (2014) on the agricultural effects of predicted climate change in the northern part of Norway (65–70°N) estimates a prolonged growing season of 11–25 days in Tromsø for the period 2021–2050, compared with the 30-year normal (1961–1990). Mean temperature is also predicted to be higher. Higher mean temperatures and longer growing seasons will inevitably lead to higher accumulated day-degree sums. For coastal sites such as Tromsø, the increase in day-degree sums during the growing period is predicted to be in the range of 100–200 (Hanssen-Bauer et al., 2010). Based on the phenology data in this study, this increase in day-degree sums might still be insufficient for the full maturation of peas in this Arctic location, while peas regularly reaching “green maturation” would be more likely.

Growers at locations above the Arctic Circle often cannot establish the fields until they are close to the summer solstice due to snow cover and ground frost. An earlier start to the growing season as a result of increased spring temperatures would allow more growth to take place during high light intensity and longer 24-h photoperiods of the midnight sun period of continuous daylight (Mølmann et al., 2021). As already discussed, this study indicates that longer photoperiods and longer daily PARs could reduce the temperature sum requirement at high latitudes. It is still unclear whether increased temperatures in combination with the midnight sun could contribute to a further reduction in the heat sum requirement for the flowering and development of peas in the far north.

Longer-term scenarios predict considerably higher temperatures toward the end of the century (2071–2100), with the highest increase in the northern regions and the greatest increase in air temperature in spring and autumn (Hanssen-Bauer et al., 2009). If these predictions become reality, commercial pea production in the Arctic regions of northern Europe would appear to be a promising prospect, paving the way for the cultivation of Arctic peas.

## Data Availability Statement

The original contributions presented in the study are included in the article/Supplementary Material, further inquiries can be directed to the corresponding author/s.

## Author Contributions

UC-N was the Principal Investigator for the project who also conceived and designed the experiments, selected genotypes for the pea collection, designed and carried out the field trials, collected the phenotypic data, and drafted and finalized the manuscript, with writing contributions from all the authors. KA conceived and designed the experiments, selected genotypes for the pea collection, designed and carried out the field trials, collected the phenotypic data, and drafted and finalized the manuscript with writing contributions from all the authors. IMV conceived and designed the experiments, analyzed the data, selected genotypes for the pea collection, designed and carried out the field trials, and collected the phenotypic data. AR conceived and designed the experiments, analyzed the data, designed and carried out the field trials, and collected the phenotypic data. JBM analyzed the data. SKR conceived and designed the experiments, designed and carried out the field trials, and collected the phenotypic data. MN analyzed the data. EW carried out the field trials and collected the phenotypic data. PP conceived and designed the experiments, designed and carried out the field trials and collected the phenotypic data. GP conceived and designed the experiments and selected genotypes for the pea collection. MWL conceived and designed the experiments, selected genotypes for the pea collection, and drafted and finalized the manuscript with writing contributions from all the authors. All the authors discussed the results, read, and approved the final manuscript.

## Conflict of Interest

PP was employed by the company Boreal Plant Breeding Ltd. and GP was employed by the company Danish seed Savers. The remaining authors declare that the research was conducted in the absence of any commercial or financial relationships that could be construed as a potential conflict of interest.

## Publisher's Note

All claims expressed in this article are solely those of the authors and do not necessarily represent those of their affiliated organizations, or those of the publisher, the editors and the reviewers. Any product that may be evaluated in this article, or claim that may be made by its manufacturer, is not guaranteed or endorsed by the publisher.
